# Effects of Mobility-Enhancing Nursing Intervention in Patients with MS and Stroke: Randomised Controlled Trial

**DOI:** 10.1155/2015/785497

**Published:** 2015-02-17

**Authors:** Lorenz Imhof, Susanne Suter-Riederer, Jürg Kesselring

**Affiliations:** ^1^Institute of Nursing, Zurich University of Applied Sciences (ZHAW), 8401 Winterthur, Switzerland; ^2^Rehabilitation Clinics Valens, 7317 Valens, Switzerland

## Abstract

*Background*. Multiple sclerosis (MS) or stroke causes functional impairment which can have a major impact on patients' life.* Objectives*. This RCT investigated the effect of a new nursing intervention (Mobility Enhancing Nursing Intervention—MFP) designed to improve rehabilitation outcomes.* Method*. The study took place in a rehabilitation clinic in Switzerland. One hundred forty participants diagnosed with MS, stroke, and brain injuries were randomly assigned to control group (CG = standard care) or intervention group (IG). The IG combined standard care with 30 days of MFB. MFP placed patients on a mattress on the floor and used tactile-kinaesthetic stimulation to increase spatial orientation and independency. Outcomes were functionality (Extended Barthel Index, EBI), quality of life (WHOQoL), and fall-related self-efficacy (FES-I).* Results*. There was a significant main effect of the intervention on functionality (EBI-diff/day mean = 0.30, versus mean = 0.16, *P* = 0.008). There was also a significant main effect on QoL (WHOQoL-diff mean = 13.8, versus mean = 5.4, *P* = 0.046). No significant effect was observed on fall-related self-efficacy.* Conclusions*. The positive effect of MFP on rehabilitation outcomes and quality of life suggests that this specialized nursing intervention could become an effective part of rehabilitation programs. The study was approved by the Ethics Committee of St. Gallen (KEK-SG Nr. 09/021) and registered at ClinicalTrial.gov NCT02198599.

## 1. Background and Objectives

Neurological conditions like multiple sclerosis (MS) or stroke cause functional impairment and handicap which can have a major impact on patient quality of life. Despite symptoms and disabilities varying based on underlying causes and individual manifestations, a major impairment in sensory function, orientation, and mobility commonly presents great challenges to the affected persons, their families, and healthcare providers. Most patients wish to live independently despite condition-related restrictions. Therefore, treatment focuses on symptom management and the prevention of acute episodes and disability [1]. Low-intensity rehabilitation improves quality of life, overall health, activity, and participation in social life [2, 3].

To enable individuals to reach their goal of living independently at home, specialised rehabilitation clinics provide care by multidisciplinary programs. The aim is to improve functionality, expand kinaesthetic competence in order to increase compensation of limitations, and improve quality of life [4, 5].

Although specialised rehabilitation nursing care is an important aspect of rehabilitation programs, few studies have investigated the contribution on the effect of specialised rehabilitation nursing care within rehabilitation programs.

## 2. Methods

### 2.1. Trial Design

Through expertise, experience, and careful observations of clinical practice, nurses of a rehabilitation centre in Switzerland developed and refined a standardised intervention (mobility-enhancing nursing intervention (MFP)) to specifically enhance patient safety, body perception, kinaesthetic competence, mobility, and functionality, as well as to reduce the burdens of care on relatives [6, 7]. MFP focuses on individual self-management and is an integrated part of the nursing care in the patients' daily life at the centre. In this randomised controlled trial it was hypothesised that MFP would increase independence, quality of life, and fall-related self-efficacy in patients with MS, stroke, and brain injuries. The study was approved by the Ethics Committee of the Canton of St. Gallen (Ref. KEK-SG Nr. 09/021) and registered at ClinicalTrial.gov NCT02198599.

### 2.2. Participants

The study was conducted in a specialised neurorehabilitation clinic in the German-speaking part of Switzerland. All patients entering the clinic (from 2011 to 2013) were screened by registered nurses with special training for the following inclusion criteria: (1) diagnosed with MS, stroke, or brain injuries; (2) German-speaking; (3) aged 18 and older; and (4) cognitively able to give written consent.

### 2.3. Intervention

MFP is a nursing intervention based on the assumption that learning takes place through movement [8]. Human development is seen to be based on the interaction between a person and the environment. Tactile-kinaesthetic perception is important with regard to the way that the environment is perceived and fundamental to the development, organisation, and reorganisation of the brain. Thus, tactile-kinaesthetic stimulation is used by nurses during the mobilisation process. It was hypothesised that the way the patients perceived themselves, their environment, their body position changes, and hence cognitive-linguistic, social, emotional, and motor behaviour would be enhanced. For these purposes, the patients' mattresses were placed on the floor, which enabled the patients to explore their environment safely without the risk of falling. Additionally, the patients' environment was arranged in accordance with a nursing assessment pertaining to the patients' impairment and abilities, their goals in terms of improved mobility, and the mobility they would require in order to live at home as independently as possible. Initially, most patients favoured a specific side to get up. The goal of the intervention was to teach the patients to get up step by step and to move independently over both sides. Compared to standard mobilisation procedure from a bed which uses gravity, MFP care enables patients to overcome gravity which requires tailored support. Hence this technique improves the spatial orientation of the patients. Constant tactile-kinaesthetic stimulation was applied by guiding the person from her current position, for example, standing, laying, or sitting in a wheelchair, to the floor. With successive gestures and position changes, the person was guided back into the original position using kinaesthetic [9]. In order to implement the intervention, all nurses on the wards received training in two units of 3 and 5 days and ongoing clinical training (2–4 hours a month) on kinaesthetic principles. These principles deal with interaction, functional anatomy, human movement and function, effort, and environment [9]. The main goal of the nurses' training was to get familiar with the specific steps and the principles of kinaesthetic support of movement. In the intervention group, MFP was applied during 30 days in addition to the standard rehabilitation program in the control group, which was provided by physicians, physiotherapists, occupational therapists, and standard nurses.

### 2.4. Outcomes

Data were collected before randomisation (T0), after 15 days (T1) and at discharge (T2). Demographic data included age, gender, marital status, and living arrangements. Further medical data were collected (diagnosis, length of stay, and discharge destination).

### 2.5. Outcome Variables

Functional health is an important patient outcome of nursing care [10]. Therefore, the primary outcome was functionality. To measure functionality, the Extended Barthel Index (EBI), a validated and common instrument in rehabilitation settings, was used [11, 12]. The EBI includes 16 items that are rated on a 4- and 5-point Likert scale (not possible, with support of a person, with low support, with facilities, and independent). A score of 64 points indicates maximum independence [13].

Secondary outcome variables were the need for nursing care after discharge, quality of life, and fall-related self-efficacy. The need for nursing care was measured with the Self-Care Index (SPI) which is based on nine functional items and one cognitive item [14, 15]. These items are part of the clinical Assessment for Acute Care Instrument (ePA_AC), used in the Swiss rehabilitation setting to plan necessary nursing care [16]. The maximum SPI score of 40 points means complete independent living possibilities. Scores below 32 points show a need for nursing care after discharge [17].

Quality of life was measured using the German version of the WHOQoL-Bref. The instrument includes 26 items that are rated on a 5-point Likert scale (very poor to very good, very dissatisfied to very satisfied, not at all to an extreme amount, not at all to extremely, and never to always). The WHOQoL-Bref yields a score for general quality of life in each of the four domains, physical, psychological, social, and environmental, with a score of 100 indicating maximum quality of life. Internal consistency for the subscales ranges between an alpha of 0.70 and 0.86 [18].

To measure fall-related self-efficacy and fear of falls, the seven-item short version of the Fall Efficacy Scale (FES-I) was used [19]. The FES-I is a well established and validated (Cronbach's alpha was 0.85 in a sample of MS patients) instrument with a 4-point Likert scale [20]. Scores range from 7 to 28. A higher score is synonymous with more fear of falls and less self-efficacy [21, 22].

### 2.6. Sample Size

The sample size was calculated based on data obtained during a pilot study which showed a clinically relevant medium effect size of 0.54 for EBI. The power was set at 0.8, and alpha was set at 0.05 resulting in a required sample size of 126 in total or 63 per group for a 1 : 1 allocation. Allowances were then made for an attrition rate of 30%; hence the target was to recruit 162 participants or 81 per group. As the attrition rate was well below the estimated rate of 30%, recruitment was stopped when a sample size of 140 was achieved.

### 2.7. Randomisation and Blinding

Participants were then randomly allocated in blocks of ten to intervention or control group (1 : 1) using a computer-generated list for random numbers. The randomisation process was conducted by a person not involved in the study. A research assistant that was not involved in the delivery of the intervention collected the data for outcome measures. Due to the nature of the intervention, blinding was not possible.

### 2.8. Statistical Analysis

Data were analysed using SPSS version 19 (SPSS, Inc., Chicago, IL). For all outcome measures (EBI, WHOQoL, and FES-I) changes between baseline (T0) and discharge (T2) were calculated. Since length of stay showed a wide range among patients, changes for the EBI were calculated as mean changes per day (EBI-diff/day). To determine the demand for nursing care after discharge the percentage of participants with a Self-Care Index (SPI) below 32 points was calculated. In order to test the hypotheses, the changes were compared between the intervention groups (intervention and control) and between two diagnostic groups (MS and stroke). A two-way between-group covariance analysis was conducted to test the impact of the intervention on the rehabilitation outcomes. Both the EBI score and the WHOQoL index at baseline were introduced as covariates. To test the effect on fall-related self-efficacy (FES-I), a two-sided Student's *t*-test was performed. Analysis will be by intention to treat as per protocol.

## 3. Results

### 3.1. Participants

**Figure 1:**
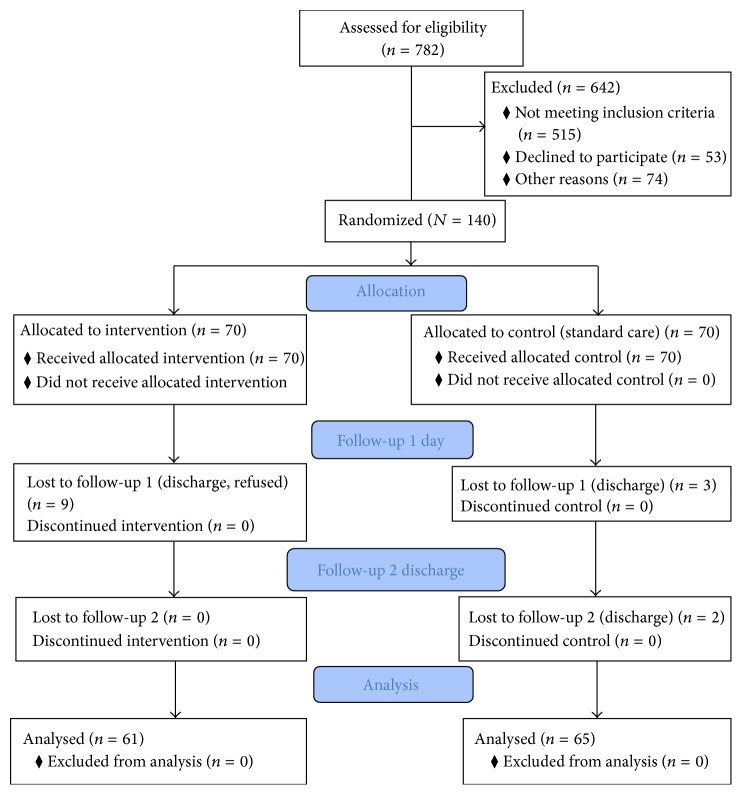
Recruitment process.

Between April 2011 and March 2013, 782 patients were screened, 140 of whom were recruited and randomly assigned to the intervention group (*n* = 70) or the control group (*n* = 70). Results are based on 126 participants included in final analysis (see Figure 1).

### 3.2. Baseline and Medical Data

The characteristics of the two study groups were comparable at baseline (Table 1). The mean age of the participants was 62 years (SD ± 13.6) and 49% were female and 95% (*n* = 133) lived at home prior to the clinic stay. Patients were diagnosed with MS (59%), stroke (54%), and traumatic brain injury (*n* = 5). Due to the small number of patients with brain injuries, this group was not included in the analysis. The mean EBI at baseline was 41.5 points (SD ± 10.7). There was no significant difference between intervention and control group at baseline (mean = 40.6, SD ± 9.6 versus mean = 42.4, SD ± 11.79; *P* = 0.35). Quality of life was high (mean 52.9 SD ± 24.9), approximately 8 points above the German norm rate for MS patients [18]. There was no significant difference at baseline between the intervention and control group (mean = 49.6, SD ± 25.4 versus mean = 56.2, SD ± 24.1; *P* = 0.12). After rehabilitation 83% (*n* = 116) returned home. The average length of stay was 36 days (SD ± 21.6). The groups were well balanced for demographic and clinical characteristics at baseline T0. Dropout rate was 10%.

### 3.3. Outcomes

A significant main effect of the intervention was observed on functionality (EBI-diff/day: *F*(1,125) = 7.158, *P* = 0.008) (Table 2). The effect size was medium (partial eta squared = 0.056). The mean increase in the EBI-diff/day score was 0.14 points higher (95% CI = 0.04–0.24, *P* = 0.006) in the intervention group (mean = 0.30, SD ± 0.31) than in the control group (mean = 0.16, SD ± 0.24). A significant main effect of the diagnostic groups was also observed (*F*(1,125) = 9.401, *P* = 0.003). The effect size was medium (partial eta squared = 0.072). The mean increase in the EBI-diff/day score was 0.24 points higher (CI = 0.14–0.33, *P* = 0.000) in the stroke group (mean 0.33, SD ± 0.30) compared with MS group (mean = 0.10, SD ± 0.20). The analysis showed no statistically significant interaction between the intervention and diagnostic group (*F*(1,125) = 0.222, *P* = 0.638).

There was a significant effect of the EBI covariate at entry T0 (*F*(1,125) = 4.671, *P* = 0.034). The effect size was medium (partial eta squared = 0.037). The test for the second outcome, quality of life, showed a significant main effect of the intervention on WHOQoL-diff (*F*(1,116) = 4.06, *P* = 0.046). The effect size was medium (partial eta squared 0.034). The mean increase in WHOQoL was 8.4 points higher (CI 0.14–16.6, *P* = 0.045) in the intervention group (mean = 13.8, SD ± 19.6) than in the control group (mean = 5.4, SD ± 25). The analysis showed no statistically significant interaction between the intervention and diagnostic group (*F*(1,125) = 0.222, *P* = 0.638). No significant effect on fall-related self-efficacy (FES-I) between T0 and discharge was observed. There was also a significant difference in the Self-Care Index (SPI). The percentage of participants that remained below an index of 32 at discharge, indicating the need for nursing care after discharge, was significantly lower in the IG than in the CG (52.9% versus 80.6%, *P* = 0.001).

## 4. Discussion

The results of this first study investigating the effect of MFP carried out by specially trained nurses show that the intervention is effective to enhance mobility and quality of life of individuals with MS and stroke. The hypothesis that MFP enhances clinically relevant rehabilitation processes was confirmed by this study. The results strengthen the concept to integrate functional training into habitual daily routines [23], to create special, individualized context in order to speed up the rehabilitation process, and to produce sustainable effects on the patients' functionality.

To the best of our knowledge, little is known about the sensitivity to changes in either the Extended Barthel Index or the Barthel Index [24]. However, one can assume that a one-point change on a 4-point scale is clinically meaningful, because the graduations of the EBI are quite large. At discharge the detected change in the IG is almost double (13 points) the CG (6.8) and is presumably clinically significant for the patients.

We assume an increase in WHOQoL-Bref of 13.8 points is also meaningful. This increase is higher than found in at least one other study [25]. We propose that this change will have a clinically significant impact on the subjective patients' perception of quality of life. Even de Souza et al. [26] did not find a correlation between the level of disability and quality of life; further research is needed to determine how enhanced mobility influences quality of life.

We were surprised that the change in fear of falling and self-efficacy was not significant in this study. In interviews performed during data collection, patients explained how their new ways of moving around had made them aware of risks. During the long adaptation process, they experienced a wide range of emotions, ranging from frustration to success. These heterogeneous processes and the short observation period stretching slightly over a month could have led to this nonsignificant result. Rosén et al. [27] mentioned another difficulty with FES, as some patients may have difficulty estimating their self-confidence without performing specified activities. Further research is needed to determine how patients estimate their self-efficacy during rehabilitation process.

The clinical significance of the results was also shown by the difference of the SPI, since the SPI is used in Swiss rehabilitation clinics to indicate the need of nursing care. The increase in self-care ability during the hospitalisation in the intervention group was twice that of the control group. Among the control group participants, 80.6% of the individuals showed the need for additional nursing care after discharge. This percentage was 27.7% lower in the intervention group, where only 52.9% showed the same need for nursing care. Therefore, it can be assumed that MFP has a positive effect on health care services, especially nursing home care. Additional research is needed that looks at rehabilitation outcomes related to health care utilisation after discharge.

To fully appreciate the clinical significance of the MFP intervention, we explored the qualitative statements of the patients themselves. A study investigated the lived experience of patients with MFP. These results show a heterogeneous and individual experience and evaluation of the personal benefit and significance. Further research is needed to explore these patient-centered changes.

Nurses are well aware that patients dealing with the aftermath of a stroke or in remission of MS need comprehensive treatments and care that are not limited to physiological retraining. MFP encompasses a series of actions performed several times during the day. The intervention is guided by principles of patient centeredness, negotiation of shared goals, and a perspective that includes family members. On one hand, this personalised, close contact may challenge both patient and nurse alike, especially during the intervention. On the other hand, it fosters a caring nursing relationship, which could explain why a considerable increase in quality of life was observed at discharge. The higher increase in the self-performance of activities of daily living combined with the higher increase in quality of life shows that MFP has the potential to boost the rehabilitation effect on a statistically and clinically significant level.

## 5. Limitations

The generalisation of the study findings is limited because of the fact that the design did not allow for blinding data collection.

At the beginning of a rehabilitation process, people with brain injuries often were cognitively not able to give written consent. Due to the decision of the Ethics Committee, this group was not eligible to take part in this study.

## 6. Conclusion

Further research is needed to investigate the effect of MFP and how nursing interventions combined with rehabilitative therapies contribute to the multiprofessional rehabilitation success. Since MFP is only possible through specially trained nurses, it is necessary to further develop the role of rehabilitation nurses and to standardise patient-centred interventions such as MFP as a basis for further research. MFP certainly influences the nurse-patient relationship. Patient centeredness and empowerment to carry out everyday tasks are demanding challenges for both patients and nurses. The actual experiences of the patients during the intervention should therefore be investigated qualitatively, as the results from such studies could provide more insight into the basic nursing processes involved.

## Figures and Tables

**Table 1:** Baseline characteristics and medical data.

	Intervention group (IG) *n* = 70	Control group (CG) *n* = 70	*P* value
Age in years	61.8 (14.5)	62.9 (12.7)	0.625
Female	32 (45.7%)	36 (52.9%)	0.499
Diagnosis			0.578
SHT	2 (2.9%)	3 (4.3%)	
CVI	41 (58.6%)	35 (50%)	
MS	27 (45.8%)	32 (54.2%)	
EBI score	40.7 (9.6)	42.4 (11.7)	0.349
EBI stroke	36.4 (6.6)	36.3 (8.6)	0.943
EBI MS	47.3 (9.9)	49 (11.7)	0.570
WHOQoL global	49.6 (25.4)	56.2 (24.1)	0.124
Fall-related efficacy	12.7 (4.8)	13.5 (5.1)	0.366
Self-Care Index (SPI)	28.5 (6.4)	30.0 (6.8)	0.094
Length of stay	39 (24.1)	34.3 (18.58)	0.192
Discharge destination			0.908
Home	58 (84.1%)	58 (82.9%)	
Institution	9 (13%)	9 (12.9%)	
Hospital	2 (2.9%)	3 (4.3%)	

Data are mean (SD) or *n* (%), unless otherwise stated.

**Table 2:** Results difference in scores between T0 and discharge.

Variable	Score-diff		Test statistic	95% confidence interval	*P* value
	IG (*n* = 61)	CG (*n* = 65)			
EBI-diff/day	0.3 (0.3)	0.16 (0.2)	*F* = 7.158	0.04–0.24	0.006
WHOQoL-diff (global)	13.8 (19.6)	5.4 (25)	*F* = 4.06	0.14–16.6	0.046
FES-I-diff	2.4 (4.2)	2.8 (5.3)	*t* = −0.4		0.773
SPI-diff	7.5 (5.9)	3.1 (6.4)	*F* = 16.3	2.3–6.6	0.000

Data are mean (SD) and probability (95% confidence interval).
